# Mining and characterization of novel EST-SSR markers of *Parrotia subaequalis* (Hamamelidaceae) from the first Illumina-based transcriptome datasets

**DOI:** 10.1371/journal.pone.0215874

**Published:** 2019-05-06

**Authors:** Yunyan Zhang, Mengyuan Zhang, Yimin Hu, Xin Zhuang, Wuqin Xu, Pengfu Li, Zhongsheng Wang

**Affiliations:** 1 College of Life Sciences, Nanjing University, Nanjing, China; 2 Anhui Academy of Forestry, Hefei, China; 3 Key Laboratory of Conservation Biology for Endangered Wildlife of the Ministry of Education, and Laboratory of Systematic and Evolutionary Botany and Biodiversity, College of Life Sciences, Zhejiang University, Hangzhou, China; Institute for Biological Research, SERBIA

## Abstract

*Parrotia subaequalis* is an endangered Tertiary relict tree from eastern China. Despite its important ecological and horticultural value, no transcriptomic data and limited molecular markers are currently available in this species. In this study, we first performed high-throughput transcriptome sequencing of two individuals representing the northernmost (TX) and southernmost (SJD) population of *P*. *subaequalis* on the Illumina HiSeq 2500 platform. We gathered a total of 69,135 unigenes for *P*. *subaequalis* (TX) and 84,009 unigenes for *P*. *subaequalis* (SJD). From two unigenes datasets, 497 candidate polymorphic novel expressed sequence tag-simple sequence repeats (EST-SSRs) were identified using CandiSSR. Among these repeats, di-nucleotide repeats were the most abundant repeat type (62.78%) followed by tri-, tetra- and hexa-nucleotide repeats. We then randomly selected 54 primer pairs for polymorphism validation, of which 27 (50%) were successfully amplified and showed polymorphisms in 96 individuals from six natural populations of *P*. *subaequalis*. The average number of alleles per locus and the polymorphism information content values were 3.70 and 0.343; the average observed and expected heterozygosity were 0.378 and 0.394. A relatively high level of genetic diversity (*H*_T_ = 0.393) and genetic differentiation level (*F*_ST_ = 0.171) were surveyed, indicating *P*. *subaequalis* maintained high levels of species diversity in the long-term evolutionary history. Additionally, a high level of cross-transferability (92.59%) was displayed in five congeneric Hamamelidaceae species. Therefore, these new transcriptomic data and novel polymorphic EST-SSR markers will pinpoint genetic resources and facilitate future studies on population genetics and molecular breeding of *P*. *subaequalis* and other Hamamelidaceae species.

## Introduction

*Parrotia subaequalis* (H.T. Chang) R.M. Hao & H.T. Wei, the focal species of our study, is a member of the genus *Parrotia* C. A. Mey. in the Hamamelidaceae family. This species is a diploid (2*n* = 2*x* = 24) deciduous tree characterized by unique exfoliating bark, obovate leaves in green, yellow, red or purple, and distinct apetalous bisexual flowers [1, 2]. Therefore, *P*. *subaequalis* is widely cultivated as a horticultural and ornamental tree in North America, Europe and East Asia [3, 4]. However, the natural population size of the wild *P*. *subaequalis* has sharply declined due to its narrow geographic distributions in disjunct montane ecosystems of eastern China, serious habitat destruction and the species’ alternate-year fruit production [5, 6]. Additionally, as an endangered Tertiary relict tree, *P*. *subaequalis* is categorized as ‘extremely endangered’ by the International Union for Conservation of Nature (IUCN) [7] and the Chinese Plant Red Book (Grade I Key Protected Wild Plants) [8]. Thus, collection of the wild germplasm resources, plant breeding, and improvement of genetic variability of *P*. *subaequalis* has been attracting increasing amounts of attention from cultivators and researchers because of its high value in gardening applications and extant endangered survival.

Currently, molecular markers are recognized as a reliable and indispensable approach in studies of plant genetics and breeding. Specifically, molecular markers such as randomly amplified polymorphic DNAs (RAPDs), amplified fragment length polymorphisms (AFLPs), inter-simple sequence repeats (ISSRs), and simple sequence repeats or microsatellites (SSRs), are widely used for genetic diversity assessment, gene mapping, marker assisted selection and molecular breeding [9–11]. Compared with other types of molecular markers, SSRs have many advantages in abundance, hypervariability, codominant inheritance and extensive genomic and transcriptomic coverage [12]. Based on the location of the original sequences used to identify simple repeats, SSRs can be divided into genomic SSRs (gSSRs) and expressed sequence tag-simple sequence repeats (EST-SSRs). EST-SSR markers are widely used to investigate the population genetic diversity, marker-assisted selection and molecular breeding because of their higher possibility of being functionally associated with important traits or pathways and higher levels of transferability to related species as compared to gSSRs [13–15]. In addition, a series of bioinformatics tools have been developed for automated SSR discovery and marker development, such as CandiSSR, which help users to efficiently identify candidate polymorphic SSRs (PolySSRs) from transcriptome datasets or multiple assembled genome sequences rather than in a traditional time-consuming and labor-intensive way [16]. Moreover, in the last decade, with the advent of high-throughput next-generation sequencing (NGS) technologies, including 454 Life Science GSFLX Titanium and the Illumina platform, we can have access to the abundant genetic resources of the species of interest in a rapid and cost-effective way [17–19]. The transcriptome refers to the complete set and quantity of transcripts in a cell at a specific developmental stage or under a physiological condition. NGS-derived transcriptome sequencing produces large EST datasets that are exploited for molecular marker development, novel gene identification and population genetic research related to adaptive traits and pathways [20, 21].

To date, there remains a lack of available transcriptomic databases of *P*. *subaequalis* and the previously studied types of molecular markers developed for *P*. *subaequalis* merely includes ISSRs and gSSRs [6, 22]. Thus, it is imperative to enlarge the transcriptomic resources for conservation and marker-assisted breeding of *P*. *subaequalis*. In this study, we first sequenced the transcriptomes of two individuals of *P*. *subaequalis* from the northern- and southernmost populations on the Illumina HiSeq 2500 platform. The objectives of our study were to (1) provide transcriptomic information for these two *P*. *subaequalis* transcriptomes, (2) undertake the mining and characterization of novel polymorphic EST-SSR markers for *P*. *subaequalis* based on the two transcriptome datasets, and (3) perform the assessment of genetic variation in six natural populations by EST-SSR markers and their cross-species transferability.

## Materials and methods

### Plant samples, RNA, and DNA isolation

**Fig 1:**
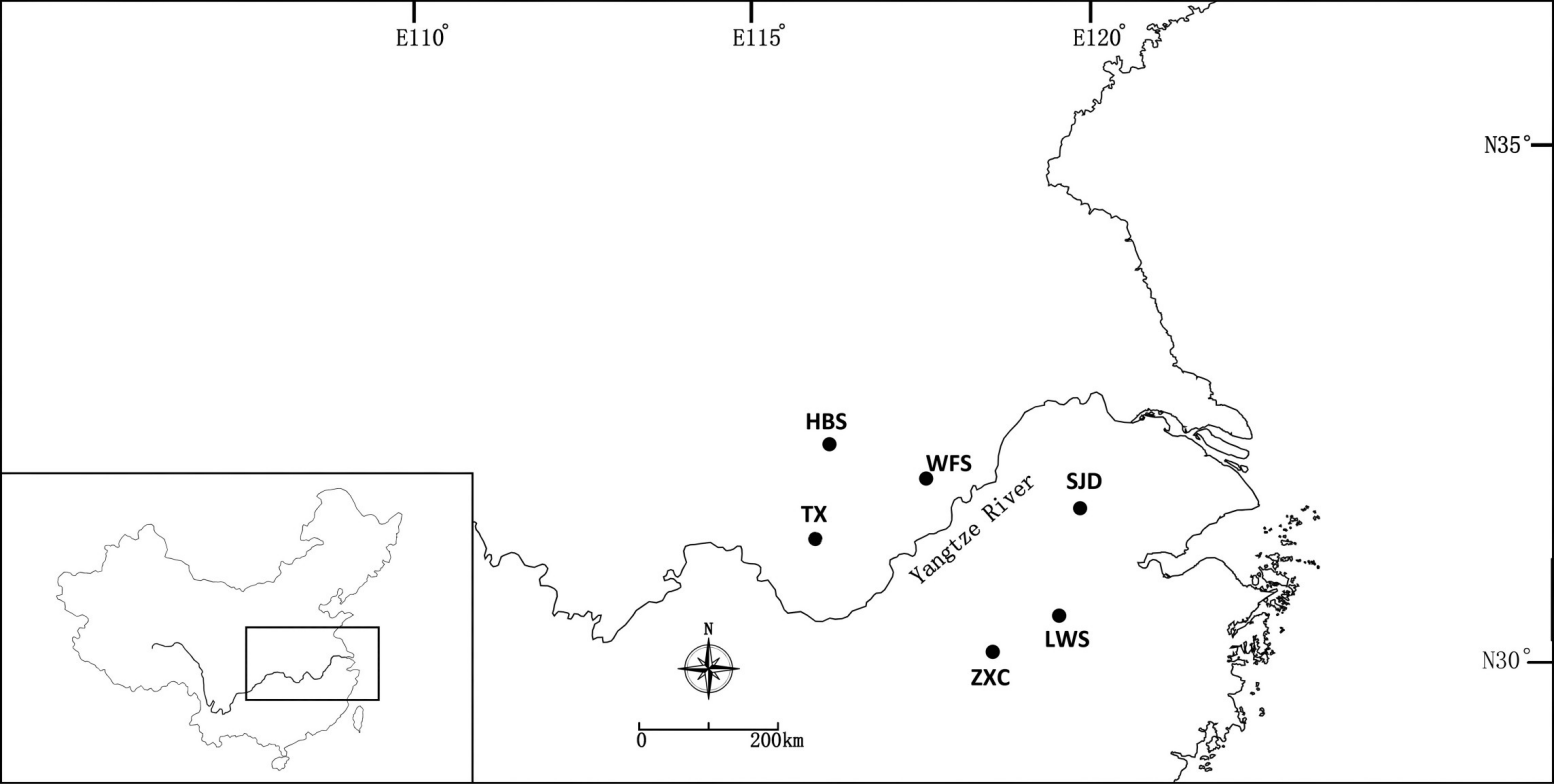
The distribution range of six natural populations of *Parrotia subaequalis* in China.

For RNA sequencing, the young and fresh leaves of two individuals of *P*. *subaequalis* were collected from two natural populations: TX in Anhui Province and SJD in Jiangsu Province (China) (Fig 1 and S1 Table), respectively. Our field work in TX population was under the authority of Tianxia Mountain Landscape Management Administration; And Shanjuan Cave Scenic Spot Management Administration gave the permission of our collection in SJD population. The leaves were chosen to represent the northern- and southernmost distribution of *P*. *subaequalis*. Before RNA extraction, all samples were immediately frozen in liquid nitrogen and stored at –80°C. Total RNA for each individual was extracted using TRIzol Reagent (Invitrogen Life Technologies, Carlsbad, California, USA) and treated with DNase (TakaRa Bio, Shuzo, Kyoto, Japan) following the manufacturer’s instructions. The integrity of the RNA was evaluated by agarose gel electrophoresis and validated using an Agilent 2100 Bioanalyzer (Agilent Technologies, Santa Clara, CA, USA). The concentration of RNA was measured using a NanoDrop LITE spectrophotometer (Thermo Fisher Scientific, Wilmington, DE, USA). To evaluate the polymorphisms of the EST-SSR markers developed from our transcriptome datasets and analyze the population genetic diversity of *P*. *subaequalis*, we collected samples from a total of 96 individuals of *P*. *subaequalis* from six natural populations (16 individuals per population) in China, including Shanjuan Cave (SJD), Mt. Huangbo (HBS), Mt. Tianxia (TX), Zhuxian Village (ZXC), Mt. Wangfo (WFS) and Mt. Longwang (LWS). The field studies in these locations were under the authority of the Shanjuan Cave Scenic Spot Management Administration, Huangbo Mountain Landscape Management Administration, Tianxia Mountain Landscape Management Administration, Zhuxian Village Committee, Wangfo Mountain Landscape Management Administration, and Longwang Mountain Landscape Management Administration, respectively. In addition, five related species in Hamamelidaceae (*Parrotia persica*, *Parrotiopsis jacquemontana*, *Sycopsis sinensis*, *Distylium racemosum*, and *Hamamelis virginiana*; S1 Table) were further selected for tests of cross-amplification of the polymorphic EST-SSR markers; for each species, five accessions were used. No specific permissions were required for these species’ collection, for they didn’t belong to the endangered or protected species. Representative voucher specimens of all plant materials were deposited in the Herbarium of Zhejiang University (HZU). Total genomic DNA was extracted from silica gel-dried leaves with Plant DNAzol Reagent (Invitrogen) following the manufacturer’s protocol. The quality of DNA was examined on 0.8% agarose gels stained with 1×GelRed (Biotium) and the concentration was checked using a NanoDrop LITE spectrophotometer (Thermo Fisher Scientific, Wilmington, DE, USA).

### Transcriptome sequencing, *de novo* assembly and annotation

Two next-generation sequencing (NGS) cDNA libraries were normalized using a NEBNext UltraTM RNA Library Prep Kit for Illumina (New England Biolabs, MA, USA) [23]. The mRNAs of each sample were purified and enriched using poly-T oligo-attached magnetic beads. The two cDNA libraries were then pooled together and sequenced in one lane of the HiSeq 2500 platform (Illumina Inc., San Diego, California, USA) at Beijing Genomics Institute (BGI, Shenzhen, China). The base calling and quality value calculations were performed using Illumina GA Pipeline version 1.6. After filtering the adaptor contamination and low-quality reads by Trimmomatic [24], the clean reads were assembled into transcripts using Trinity version 2.5 with the default parameters [25]. TGICL software [26] was then used to cluster similar transcripts, which generated non-redundant transcripts defined as unigenes for two individuals of *P*. *subaequalis* (Table 1). To annotate and identify the putative function of the unigenes, these sequences were subjected to a BLAST (http://blast.ncbi.nlm.nih.gov/Blast.cgi) search with a cut off *E* value of 10^−5^ against the following databases: National Center for Biotechnology Information (NCBI) non-redundant protein sequences (Nr), Swiss-Prot protein (http://www.expasy.ch/sprot/), NCBI non-redundant nucleotide sequences (Nt), Eukaryotic Orthologous Groups of proteins (KOG) database (http://www.ncbi.nlm.nih.gov/KOG), protein sequence analysis and classification (InterPro) database (http://www.ebi.ac.uk/interpro/) and the Kyoto Encyclopedia of Genes and Genomes (KEGG) pathway database (http://www.genome.jp/kegg). In addition, gene ontology (GO) terms describing molecular functions, cellular components, and biological processes were assigned using the BLAST2GO program (B2G; http://www.blast2go.com) for further annotation of the unigenes in our study.

**Table 1:** Summary of the *de novo* assembly of two individuals of *Parrotia subaequalis*.

Category	Items	Number	
		*P*. *subaequalis* (TX)	*P*. *subaequalis* (SJD)
Raw-reads	Total raw reads	26,037,119	26,666,948
Clean reads	Total clean reads	25,448,383	26,066,749
	Q20 percentage	97.94%	98.21%
	GC percentage	46.48%	46.54%
Transcripts	Total number	117,794	145,619
	Average length (bp)	674	672
	N50 (bp)	1268	1245
Unigenes	Total number	69,135	84,009
	Average length (bp)	890	887
	N50 (bp)	1591	1602

### Mining of EST-SSR markers and primer design

The potential polymorphic EST-SSR loci of *P*. *subaequalis* were identified from our two non-redundant unigenes datasets using CandiSSR [16]. The parameters used in CandiSSR were as follows: the flanking sequence length of 100, blast e-value cutoff of 1e-10, blast identity cutoff of 95, and blast coverage cutoff of 95. For each target EST-SSR, primers were automatically designed in the pipeline based on the Primer3 package [27] with default settings: PCR product size of 100 to 300 base pairs (bp), primer length of 18–25 bp, annealing temperature between 48 and 60°C, and CG content from 40 to 60%. The OLIGO version 6.67 (Molecular Biology Insights, Inc., Cascade, Co, USA) was then used to check for hairpin structures, potential primer dimers and the occurrence of mismatch of the above designed primer pairs.

### EST-SSR polymorphism validation and transferability test

Based on the proportion of different EST-SSR repeats, we randomly chose 54 candidate polymorphic primer pairs for initial tests of amplification availability and optimal annealing temperature of each primer pair using six samples (one individual per population) of *P*. *subaequalis*. The gradient PCR amplifications were performed on a GeneAmp9700 DNA Thermal Cycler (Perkin-Elmer, Waltham, Massachusetts, USA) following the standard protocol of the AmpliTaq Gold 360 Master PCR kit (Thermofisher Biotech Company, Applied Biosystems, Foster City, California, USA) in a final volume of 15 μL, which contained 1 μL (50 ng) of genomic DNA, 7.5 μL AmpliTaq Gold 360 Master Mix, 5.5 μL of deionized water, and 0.5 μL of forward and reverse primers (10 μM). The procedure of PCR was 5 min initial denaturation at 95°C, 35 cycles of 45 s at 95°C, a temperature gradient for annealing from 48°C to 60°C for 30 s and 30 s synthesis at 72°C followed by a final 10-min extension step at 72°C and a 4°C holding temperature.

The polymorphisms of the above successfully amplified loci were screened by means of fluorescence-based genotyping using 96 individuals from six natural populations of *P*. *subaequalis*. For all loci, the 5’ end of each forward primer was tagged with one of four fluorescent dyes (FAM, ROX, HEX or TAMRA), and PCR amplifications were performed on a GeneAmp9700 DNA Thermal Cycler (Perkin-Elmer, Waltham, Massachusetts, USA) using reaction volumes of 15 μL including 1μL (50 ng) of template DNA, 7.5 μL AmpliTaq Gold 360 Master Mix, 5.5 μL of deionized water, and 0.5 μL of reverse and fluorophore-labeled forward primers (10 μM). PCRs were run following an endpoint PCR procedure with initial denaturation for 5 min at 95°C followed by 35 cycles of 95°C for 45 s, 30 s annealing at the optimal primer temperature (Table 2) and 30 s synthesis at 72°C; ending with a final 10-min extension step at 72°C and a 4°C holding temperature. PCR products were analyzed on an ABI 3730XL DNA Analyzer (Applied Biosystems, Foster City, California, USA) with GeneScan LIZ 500 as an internal reference (Applied Biosystems). Electrophoresis peaks scoring and polymorphism identification were assayed by using GeneMarker v2.2.0 (SoftGenetics, State College, Pennsylvania, USA). All primer sequences obtained from this study were submitted to GenBank (Table 2).

**Table 2:** Characteristics of the 27 polymorphic EST-SSR markers developed for *Parrotia subaequalis*.

Locus	Primer sequences (5’- 3’)	Repeat motif	Allele size range (bp)^a^	Ta (°C)	Fluorescent dye^b^	GenBank accession no.	BLAST top hit description [organism]^c^	E-value
PasE6	F: GCCAAACAACACCAACAAACC R: GTCGCCGATGGAGaGTAAGAC	(AAG)_5_	147–153	53	FAM	MK238352	─	─
PasE20	F: TGTGGTGACAAAAGACACAGT R: TGCTTGTCATACGATGATTC	(AC)_6_	184–200	48	FAM	MK238353	─	─
PasE27	F: TCTCTTCACCCATCTCCCCAT R: GTTGGGTGGGTTTCAGAGCT	(AC)_7_	172–178	51	HEX	MK238354	Zinc finger CCCH domain-containing protein 20 [*Morus notabilis*]	2E-06
PasE83	F: TGGCAGACAACGAAGGATGG R: CCATCTCGGTTGCCACTTCT	(AGC)_5_	155–167	52	HEX	MK238355	Zinc finger AN1 and C2H2 domain-containing stress-associated protein 11 [*Quercus suber*]	1E-50
PasE108	F: CTCCGTTGACCAAAACTGGAC R: CCAAAGAATCCTGCAAAGAAAGC	(AT)_6_	202–208	59	TAMRA	MK238356	─	─
PasE156	F: GCCGATCAAGATGCGGTTTC R: CGGGGCTCTTCTTCTCCATG	(ATA)_8_	193–202	59	TAMRA	MK238357	Titin like [*Actinidia chinensis* var. *chinensis*]	1E-41
PasE159	F: TACTGCAGAAGGCCATCAGC R: TGGTGAGATGGAGCTGCTTG	(ATC)_7_	163–175	53	TAMRA	MK238358	NAC transcription factor 25 [*Populus trichocarpa*]	1E-11
PasE178	F: CTAGTCCCAGCCAAACAGCA R: CATCGAGTGGCTCCAGAGTG	(CAC)_6_	123–129	53	ROX	MK238359	Eukaryotic translation initiation factor 5-like [*Quercus suber*]	1E-18
PasE180	F: GAAAGCCCACAGTGGTTCCT R: CGACTCACAACCTGCTCCTC	(CAC)_6_	152–164	49	TAMRA	MK238360	hypothetical protein B456_008G056900 [*Gossypium raimondii*]	1E-21
PasE188	F: GACCCTGCCCATCTTCTGTC R: GTGCAGTGTTCTGTCTCACG	(CAT)_6_	134–155	56	TAMRA	MK238361	─	─
PasE198	F: CGCCAAGGACAGTGATGAGT R: AAGTCGGGCCCGGAATATTC	(CCG)_6_	105–111	53	ROX	MK238362	Hypothetical protein T459_10871 [*Capsicum annuum*]	1E-04
PasE205	F: CTCCCGTACCTTCGATCACG R: TCTTCGGATGGAGGGTCACT	(CGC)_5_	132–135	52	ROX	MK238363	E3 ubiquitin-protein ligase CIP8 [*Prunus avium*]	1E-26
PasE208	F: CAGTGTGAGCTCAACGAGGT R: TCCTCGGCACTCCCTTAGAT	(CGG)_6_	164–173	56	FAM	MK238364	Hypothetical protein CDL12_15788 [*Handroanthus impetiginosus*]	1E-18
PasE218	F: TCGCTCTCCTCTGATCTGCT R: CAACCGCCATGCTTTCTCAC	(CT)_6_	116–120	56	ROX	MK238365	Hypothetical protein CICLE_v10030533mg [*Citrus clementina*]	1E-08
PasE268	F: TTGATTTCACTCCCGGCGAA R: ACTTTCTTGCCAGAGCGTGT	(GA)_7_	157–163	56	FAM	MK238366	─	─
PasE290	F: GCGAAAGATGAAGCGAAGAGG R: TCCACCATGAAACTGAGGCT	(GAA)_5_	151–160	53	TAMRA	MK238367	F-box protein SKIP22 [*Populus trichocarpa*]	1E-17
PasE300	F: GCTGGTGCTGAAGATGAGGA R: ACTCCTCTGCAACCTCCATTG	(GAT)_5_	187–190	59	HEX	MK238368	Polyprenyl synthetase [*Parasponia andersonii*]	1E-58
PasE304	F: TCCATGTAACAAGTAAGCGGCTA R: TCGTGTCTTCTCATTACTCCACA	(GAT)_7_	111–114	56	ROX	MK238369	─	─
PasE348	F: GCCGCCGATTCAAGAGATTC R: ACGATTCACCTCCGAACCTC	(TA)_6_	184–190	49	ROX	MK238370	SAC3 family protein A isoform X1 [*Prunus yedoensis* var. *nudiflora*]	2E-06
PasE368	F: AGCACAACGTACTCAACTCCT R: ACTACATACGCACCGCAGTT	(TA)_7_	155–173	57	FAM	MK238371	Hypothetical protein CCACVL1_08931 [*Corchorus capsularis*]	1E-12
PasE380	F: ACATCAATAGAGGATCGGTT R: TGTGAGCACACCAAACTATG	(TA)_8_	200–204	53	ROX	MK238372	Hypothetical protein DAPPUDRAFT_104540 [*Daphnia pulex*]	2E-56
PasE425	F: AACCCACCATCACCACCATC R: GCTCGTCTTGAAACCGCATC	(TC)_7_	151–157	53	ROX	MK238373	Protein KINESIN CHAIN-RELATED 1-like [*Olea europaea* var. *sylvestris*]	1E-04
PasE447	F: GGGTGAGGTGGAGTTAAGGC R: CTTCCGGTATTGCACCCACA	(TCG)_7_	150–156	52	FAM	MK238374	─	─
PasE452	F: GTGGTTGTGGAAAGAGAGGGT R: GTCTGCTGCTGATGCTGTTG	(TCT)_5_	175–178	56	HEX	MK238375	Auxin-responsive protein IAA26-like isoform X2 [*Ziziphus jujuba*]	1E-09
PasE480	F: TGTTGTTGTGCTGATGACTGT R: TCCCCTTAGGCTACCATGCT	(TGA)_5_	101–104	52	HEX	MK238376	Hypothetical protein AQUCO_01400195v1 [*Aquilegia coerulea*]	1E-38
PasE486	F: TGTCATGCATCACCCCAAGG R: GCCGCCATGTCAACAAAACA	(TGT)_5_	198–201	49	TAMRA	MK238377	Hypothetical protein GOBAR_DD26384 [*Gossypium barbadense*]	1E-19
PasE487	F: TGAATGGACAAAACCAGGCT R: AGGCCCCTTCAGTAAATCACT	(TTA)_5_	178–181	57	HEX	MK238378	Leucoanthocyanidin reductase [*Vaccinium ashei*]	3E-32

*Note*: ^a^ Size range values based on 96 individuals.

^b^ Forward 5’-label.

^c^ The corresponding sequences of the 27 EST-SSRs were blasted against the GenBank nonredundant database using BLASTX.

─ = not found.

In addition, transferability tests among the other five Hamamelidaceae species, i.e., five accessions each for *Parrotia persica*, *Parrotiopsis jacquemontana*, *Sycopsis sinensis*, *Distylium racemosum*, and *Hamamelis virginiana* (S1 Table) were assessed using the same PCR conditions described above. PCR products were detected using 2% agarose gels and amplification was considered successful when one clear distinct band was visible in the expected size range. GeneMarker v2.2.0 (SoftGenetics, State College, Pennsylvania, USA) was used to score the electrophoresis peaks.

### Evaluation of population genetic diversity and variation and test of bottleneck effect

The number of alleles (*A*), observed heterozygosity (*H*_o_), expected heterozygosity (*H*_e_) and polymorphism information content (PIC) were calculated for each locus and population using CERVUS v3.0 [28]. FSTAT v2.9.3 [29] were employed to estimate the following genetic diversity parameters of each locus and six natural populations of *P*. *subaequalis*: total genetic diversity for the species (*H*_T_), genetic diversity within populations (*H*_S_) and population genetic differentiation coefficients (*F*_ST_ and *G*_ST_). The frequency of null alleles and their bias on genetic diversity were evaluated based on the expectation maximization method implemented in FreeNA [30]. Deviation from Hardy-Weinberg equilibrium (HWE) for each population and linkage disequilibrium for each primer pair were tested using a Markov chain (dememorization 1,000, 100 batches, 1,000 iterations per batch) through GENEPOP v4.2 [31]. Analysis of molecular variance (AMOVA) was performed to partition the total genetic variance among and within populations using ARLEQUIN v3.11 [32].

The program BOTTLENECK v1.2.02 [33] was used to detect the population bottleneck effect (i.e. reductions in effective population size) over past or more recent time scales under three different models of microsatellite evolution (Infinite allele model, IAM; Stepwise mutation model, SMM; Two-phased model of mutation, TPM).

## Results

### *De novo* assembly of *Parrotia subaequalis* transcriptome datasets and functional annotation of unigenes

Using Illumina high-throughput RNA sequencing technology, a total of 26,037,119 and 26,666,948 raw reads (of 125 bp length) were generated for *P*. *subaequalis* (TX) and *P*. *subaequalis* (SJD), respectively. After stringent quality inspection and data filtering, 25,448,383 and 26,066,749 high-quality clean reads were obtained for *P*. *subaequalis* (TX) with 97.94% Q20 bases (base quality greater than 20) and *P*. *subaequalis* (SJD) with 98.21% Q20 bases. The total length of the clean reads was 3.62 Gb for *P*. *subaequalis* (TX) and 3.91 Gb for *P*. *subaequalis* (SJD). The GC percentage of *P*. *subaequalis* (TX) and *P*. *subaequalis* (SJD) were 46.48% and 46.54% (Table 1). The two raw sequencing datasets were uploaded to the NCBI Sequence Read Archive (SRA, https://www.ncbi.nlm.nih.gov/Traces/sra; Biosample accession SAMN10502180 for *P*. *subaequalis* (TX) and SAMN10509852 for *P*. *subaequalis* (SJD)).

**Fig 2:**
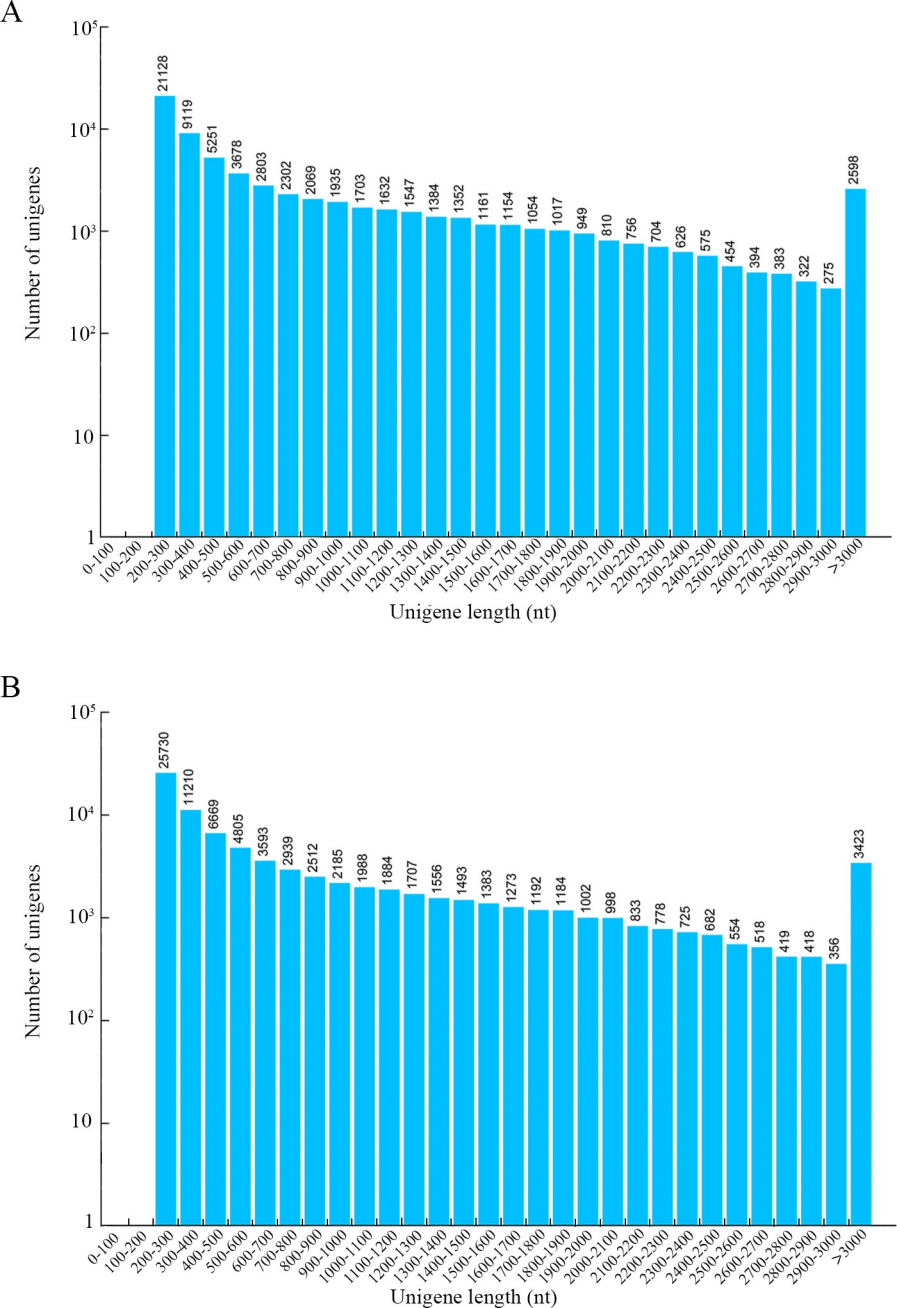
Length distribution of assembled unigenes of two *Parrotia subaequalis* transcriptomes. (A) *Parrotia subaequalis* (TX); (B) *Parrotia subaequalis* (SJD).

With the help of Trinity version 2.5, these above clean reads were *de novo* assembled into 117,794 transcripts with an average length of 674 bp and an N50 length of 1268 bp for *P*. *subaequalis* (TX) and 145,619 transcripts with an average length of 672 bp and an N50 length of 1245 bp for *P*. *subaequalis* (SJD) (Table 1). Subsequently, using TGICL software, we gathered 69,135 unigenes with an average length of 890 bp and an N50 length of 1591 bp for *P*. *subaequalis* (TX) and 84,009 unigenes with an average length of 887 bp and an N50 length of 1602 bp for *P*. *subaequalis* (SJD) (Table 1). Among the unigenes in *P*. *subaequalis* (TX), the length of 48,285 unigenes (69.84%) ranged from 300 to 1000 bp, while the other 20,850 unigenes (30.16%) were more than 1000 bp in length. Of the unigenes in *P*. *subaequalis* (SJD), 59,643 unigenes (70.00%) had a length range of 300 to1000 bp, and 24,366 unigenes (30.00%) had a length of more than 1000 bp. The length distributions of these two unigene datasets are shown in Fig 2.

Sequence similarity searching was conducted using the BLAST algorithm specifying E values of less than 10^−5^ for annotation of unigenes. For *P*. *subaequalis* (TX), of the 69,135 total unigenes, 42,978 (62.17%) were successfully annotated in at least one database and 11,958 (17.30%) were annotated in all databases. Specifically, among the annotated unigenes, 38,187 (55.24%) had hits in the Nr database, 34,760 (50.28%) in Nt, 32,331 (46.77%) in InterPro, 28,880 (41.77%) in KOG, 28,306 (40.94%) in KEGG, 25,661 (37.12%) in Swiss-Prot and 21,004 (30.38%) in GO. For *P*. *subaequalis* (SJD), we found that 51.78% (43,499) consensus sequences showed homology with sequences in the Nr database, 47.49% (39,895) in Nt, 43.51% (36,556) in InterPro, 39.29% (33,004) in KOG, 38.47% (32,322) in KEGG, 34.33% (28,840) in Swiss-Prot and 28.12% (23,624) in GO. Taken together, of the 84,009 total unigenes, 60.03% (50,429) were successfully annotated in at least one database and 15.49% (13,015) were annotated in all databases.

**Fig 3:**
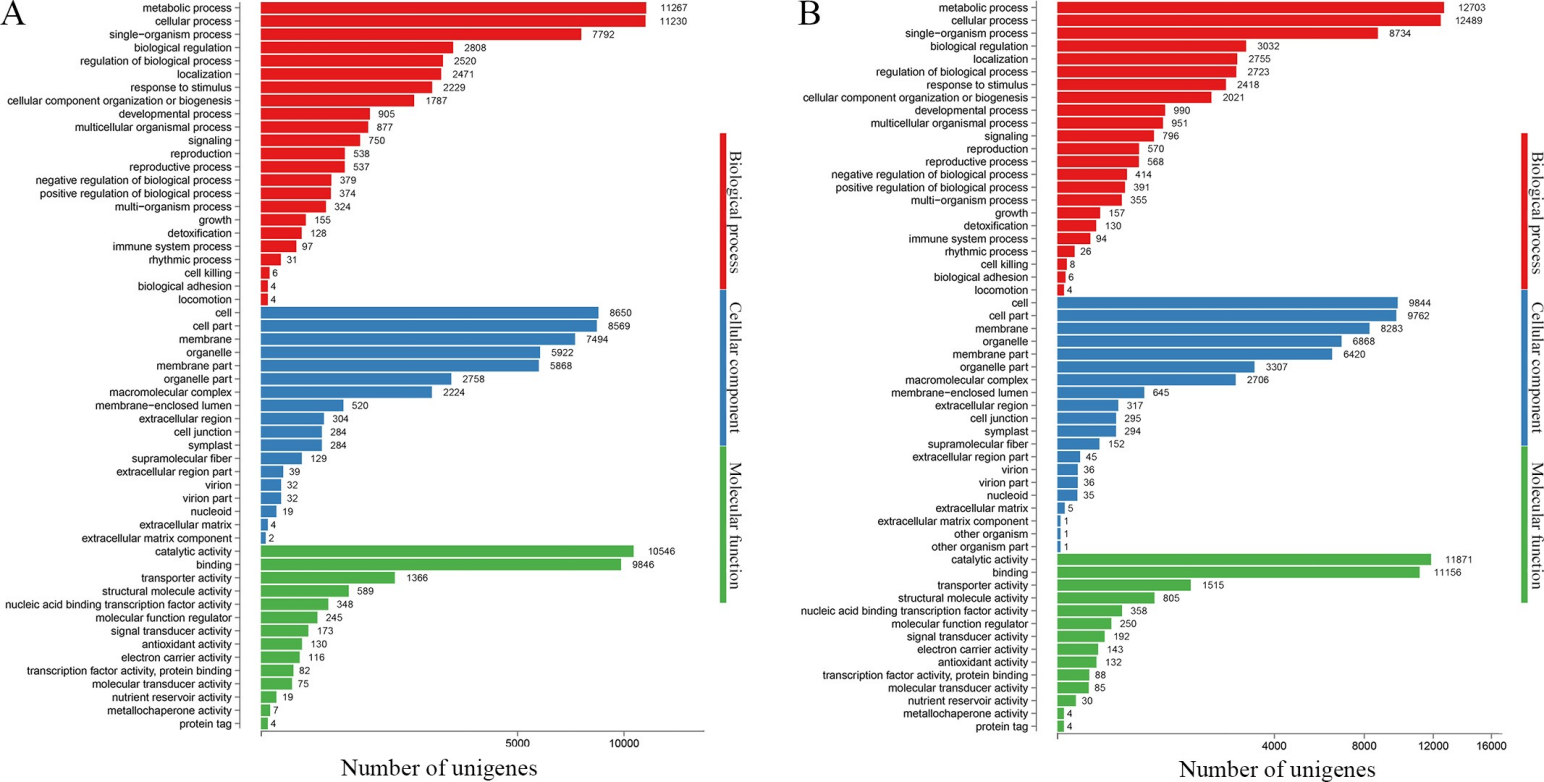
Gene ontology (GO) classification of assembled unigenes of two *Parrotia subaequalis* transcriptomes. (A) *Parrotia subaequalis* (TX); (B) *Parrotia subaequalis* (SJD).

We used the BLAST2GO program to annotate and analyze the function of unigenes in two individuals of *P*. *subaequalis* against the GO database. It comprehensively classifies the properties of genes into three categories: biological process, cellular components and molecular function. Based on sequence similarity, 21,004 unigenes (30.38%) in *P*. *subaequalis* (TX) and 23,624 unigenes (28.12%) in *P*. *subaequalis* (SJD) were classified into three main GO categories and 55 sub-categories (Fig 3 and S2 and S3 Tables). For the two individuals of *P*. *subaequalis*, the three largest sub-categories of biological process were “metabolic process”, “cellular process” and “single-organism process”; Of the cellular components, “cell”, “cell part” and “membrane” were the most highly represented terms. Among fourteen different molecular function categories, “catalytic activity” and “binding” were the two most matched classes (Fig 3 and S2 and S3 Tables).

**Fig 4:**
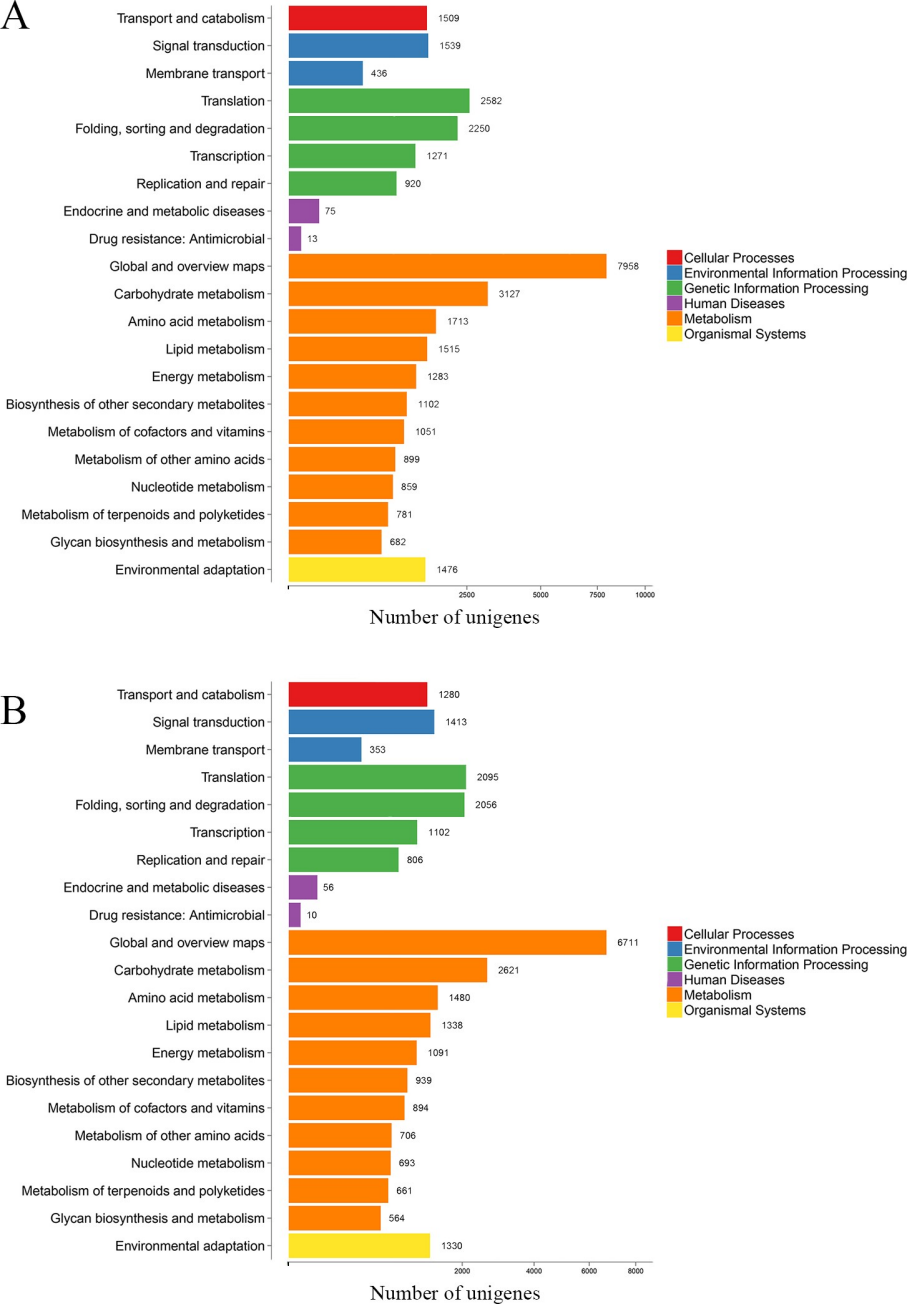
Classification map of KEGG metabolic pathways of two *Parrotia subaequalis* assemble unigenes. (A) *Parrotia subaequalis* (TX); (B) *Parrotia subaequalis* (SJD).

Furthermore, KEGG analysis was used to help us focus on the biological pathways and functions of the gene products of *P*. *subaequalis*. The results showed that 28,306 unigenes (40.94%) in *P*. *subaequalis* (TX) and 32,322 unigenes (38.47%) in *P*. *subaequalis* (SJD) were grouped into 21 biological pathways that fell under six larger groups (cellular processes, environmental information processing, genetic information processing, human disease, metabolism and organismal systems) (Fig 4 and S4 and S5 Tables). Among these 21 pathways, “global and overview maps”, “carbohydrate metabolism”, “translation”, “folding, sorting and degradation”, “amino acid metabolism” and “signal transduction” were the major biological pathways in the two individuals of *P*. *subaequalis* (Fig 4 and S4 and S5 Tables).

### Frequency and distribution of candidate polymorphic EST-SSRs

**Fig 5:**
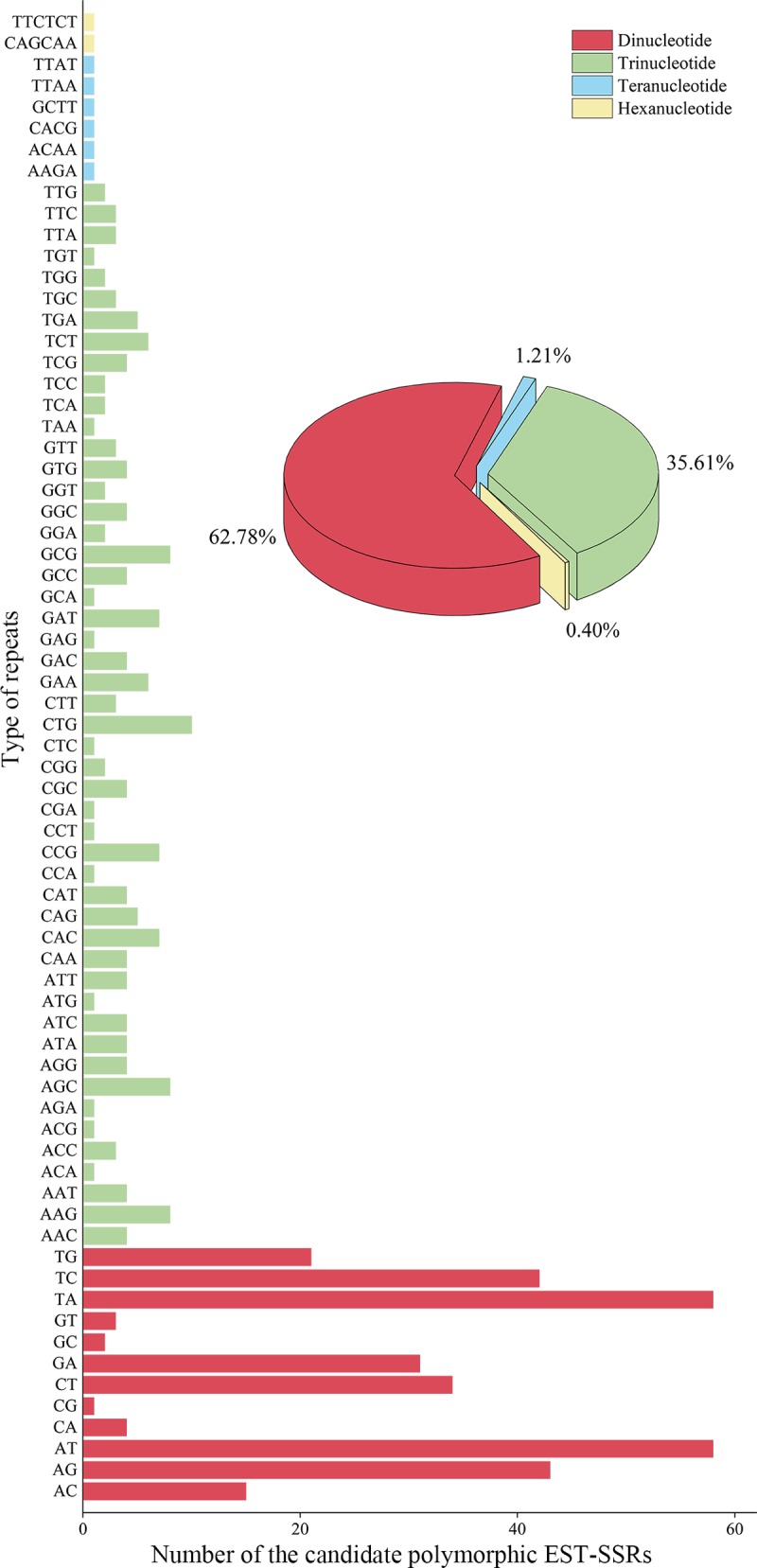
Frequency and distribution of candidate polymorphic EST-SSRs in the two *Parrotia subaequalis* transcriptomes.

Based on our two non-redundant unigenes datasets, a total of 497 candidate polymorphic EST-SSRs with an average length of 17 bp (S6 Table) were identified using CandiSSR. Of these EST-SSRs, di-nucleotide repeats (DNRs) were the most abundant repeat type (312; 62.78%), followed by tri- (TNRs; 177; 35.61%), tetra- (TTRs; 6; 1.21%) and hexa-nucleotide repeats (HNRs; 2; 0.40%) (Fig 5). Among the DNRs, AT/TA (37.18%) was quite dominant followed by AG/TC (27.24%) and CT/GA (20.83%). CTG/AAG (10.17%) was the most abundant motif for TNRs followed by AGC/GCG (9.04%) (Fig 5 and S6 Table). There were no obvious dominant motifs among the TTRs and HNRs.

### Polymorphisms and transferability assessment of EST-SSR markers

Of 497 candidate polymorphic EST-SSRs, primer pairs were designed for 488 EST-SSR loci (98.19%; S7 Table). The remaining loci were inappropriate for primer modeling or the DNA flanking sequences of these loci were too short to design primer pairs. From the 488 primer pairs, based on the proportion of different EST-SSR repeats, we randomly chose 54 primer pairs (S7 Table) for initial testing using six individuals (one sample per population) of *P*. *subaequalis* to ensure the availability and optimal annealing temperature of these primer pairs. After excluding those that gave poor amplification or produced a complex pattern with multiple bands in an initial screening, 44 primer pairs were selected for further tests of polymorphism and transferability.

To validate the polymorphisms of these 44 EST-SSR loci, fluorescence-based genotyping was performed using 96 individuals from six natural populations of *P*. *subaequalis*. Finally, 27 polymorphic primer pairs were selected for transferability and further population genetic studies, and all of these EST-SSR sequences have been deposited in GenBank with the accession numbers from MK238352 to MK238378 (Table 2). All EST-SSR markers were successfully cross-amplified and exhibited polymorphisms in five congeneric Hamamelidaceae species except for one loci (PasE380) for *Sycopsis sinensis* and two loci (PasE188 and PasE380) for *Hamamelis virginiana*, showing a transferability rate of 92.59% (Table 3).

**Table 3:** Fragment sizes detected in cross-amplification tests of the 27 EST-SSR markers in the related five species of the Hamamelidaceae group.^a^

Locus	*Sycopsis sinensis* (*N* = 5)	*Distylium racemosum* (*N* = 5)	*Hamamelis virginiana* (*N* = 5)	*Parrotiopsis jacquemontiana* (*N* = 5)	*Parrotia persica* (*N* = 5)
PasE6	180–192	153–159	183–192	180–195	153–159
PasE20	178–184	186–192	180–190	182–192	190–200
PasE27	168–176	172–180	174–182	170–178	172–178
PasE83	152–164	155–161	155–164	149–164	155–167
PasE108	200–204	198–206	200–208	200–206	200–210
PasE156	187–196	190–199	184–193	190–202	193–205
PasE159	169–175	166–175	169–178	163–175	163–175
PasE178	120–129	123–129	126–132	117–129	120–129
PasE180	155–161	164–170	152–161	155–164	152–161
PasE188	149–158	158–164	─	140–152	134–152
PasE198	102–105	105–111	102–108	108–114	105–111
PasE205	129–135	132–138	132–135	132–135	129–135
PasE208	161–170	167–176	164–173	167–173	164–173
PasE218	118–124	130–136	118–126	122–130	116–120
PasE268	167–173	165–173	163–173	173–177	165–173
PasE290	151–163	151–157	151–160	145–157	151–160
PasE300	187–190	184–193	187–193	190–199	187–190
PasE304	114–120	111–123	111–120	111–126	114–120
PasE348	176–184	174–180	180–186	178–186	184–190
PasE368	159–165	145–159	151–157	155–161	155–161
PasE380	─	198–204	─	198–202	200–204
PasE425	159–166	163–166	155–161	155–163	151–157
PasE447	150–159	147–156	153–159	147–153	150–156
PasE452	172–178	169–178	169–181	172–178	178–181
PasE480	101–107	104–110	101–110	104–107	101–104
PasE486	201–210	198–204	195–201	198–204	201–204
PasE487	178–184	181–187	178–181	178–184	178–184

*Note*: ─ = amplification failed.

^a^ Voucher and locality information are provided in S1 Table.

### Characterization of EST-SSR markers and population genetic diversity and variation

As a result, these above 27 polymorphic EST-SSRs in total yielded 100 alleles with an average of 3.70 alleles and a range of 1 to 8 alleles per locus. The polymorphism information content per locus over all populations varied from 0.060 to 0.597, and the observed and expected heterozygosity ranged from 0.063 to 0.906 and from 0.061 to 0.666 (Table 4). At the population level, average estimates of genetic diversity were medium (*H*_O_ = 0.378, *H*_E_ = 0.394), being highest in population WFS (*H*_O_ = 0.459, *H*_E_ = 0.366) and lowest in population SJD (*H*_O_ = 0.358, *H*_E_ = 0.297) (Table 4). And a high frequency of null alleles was detected in PasE188 and PasE425 (*v>*5%) for the 27 EST-SSR loci. No significant linkage disequilibrium was observed for any pair of loci. Three loci deviated significantly from HWE expectations (*P* < 0.001) in some populations (PasE20 in HBS; PasE180 in HBS and ZXC; PasE368 in HBS, TX and LWS), which might be due to the Wahlund effect of specific populations (Table 4).

**Table 4:** Genetic diversity of the 27 polymorphic EST-SSR markers in six natural populations of *Parrotia subaequalis*.^a^

	SJD (*N* = 16)				HBS (*N* = 16)				TX (*N* = 16)				ZXC (*N* = 16)				WFS (*N* = 16)				LWS (*N* = 16)				Total (*N* = 96)			
Locus	*A*	*H*_o_	*H*_e_	PIC	*A*	*H*_o_	*H*_e_	PIC	*A*	*H*_o_	*H*_e_	PIC	*A*	*H*_o_	*H*_e_	PIC	*A*	*H*_o_	*H*_e_	PIC	*A*	*H*_o_	*H*_e_	PIC	*A*	*H*_o_	*H*_e_	PIC
PasE6	2	0.125	0.121	0.110	2	0.125	0.121	0.110	3	0.375	0.401	0.334	2	0.750	0.508	0.371	2	0.313	0.417	0.323	2	0.188	0.466	0.349	3	0.313	0.530	0.422
PasE20	3	0.563	0.446	0.378	3*	1.000	0.627	0.530*	6	0.750	0.593	0.546	4	1.000	0.675	0.602	3	0.563	0.458	0.401	4	0.500	0.425	0.383	8	0.729	0.576	0.553
PasE27	3	0.813	0.534	0.412	2	0.438	0.353	0.283	3	0.438	0.433	0.354	2	0.688	0.466	0.349	2	0.250	0.315	0.258	2	0.688	0.514	0.374	3	0.552	0.470	0.368
PasE83	3	0.313	0.280	0.248	2	0.688	0.466	0.349	3	0.313	0.284	0.257	2	0.563	0.417	0.323	3	0.688	0.587	0.482	4	0.625	0.724	0.653	5	0.531	0.586	0.515
PasE108	3	0.438	0.373	0.327	2	0.250	0.226	0.195	2	0.063	0.063	0.059	1	0.000	0.000	0.000	5	0.250	0.433	0.400	3	0.188	0.179	0.166	5	0.198	0.220	0.208
PasE156	3	0.813	0.659	0.567	3	0.688	0.486	0.386	2	0.125	0.315	0.258	3	0.563	0.538	0.451	2	0.750	0.484	0.359	2	0.313	0.466	0.349	4	0.542	0.613	0.554
PasE159	3	0.688	0.542	0.416	3	1.000	0.669	0.575	3	0.813	0.546	0.419	2	0.063	0.063	0.059	3	0.563	0.522	0.450	3	1.000	0.621	0.516	4	0.688	0.591	0.500
PasE178	4	0.750	0.639	0.559	2	0.688	0.466	0.349	2	0.563	0.498	0.366	2	0.500	0.444	0.337	3	0.313	0.401	0.334	4	0.688	0.700	0.616	4	0.583	0.612	0.531
PasE180	3	0.875	0.589	0.496	5*	1.000	0.714	0.638*	3	0.938	0.647	0.551	3*	1.000	0.546	0.419*	3	0.938	0.663	0.568	3	0.688	0.579	0.496	5	0.906	0.666	0.597
PasE188	2	0.313	0.417	0.323	4	0.813	0.663	0.577	3	0.375	0.325	0.281	2	0.313	0.272	0.229	4	0.375	0.621	0.547	2	0.125	0.484	0.359	6	0.385	0.504	0.458
PasE198	1	0.000	0.000	0.000	2	0.063	0.063	0.059	1	0.000	0.000	0.000	1	0.000	0.000	0.000	2	0.125	0.121	0.110	2	0.188	0.175	0.155	3	0.063	0.061	0.060
PasE205	1	0.000	0.000	0.000	1	0.000	0.000	0.000	1	0.000	0.000	0.000	2	0.500	0.508	0.371	1	0.000	0.000	0.000	2	0.063	0.175	0.155	2	0.094	0.162	0.148
PasE208	3	0.250	0.232	0.210	3	0.688	0.556	0.447	4	0.875	0.679	0.607	2	0.500	0.387	0.305	2	0.375	0.484	0.359	3	0.375	0.476	0.398	4	0.510	0.578	0.515
PasE218	2	0.250	0.226	0.195	2	0.938	0.514	0.374	2	0.625	0.444	0.337	2	0.750	0.484	0.359	3	0.750	0.524	0.428	2	0.375	0.315	0.258	3	0.615	0.434	0.347
PasE268	3	0.313	0.280	0.248	2	0.625	0.444	0.337	2	0.375	0.387	0.305	2	0.500	0.508	0.371	2	0.438	0.498	0.366	2	0.750	0.484	0.359	3	0.500	0.486	0.385
PasE290	2	0.063	0.063	0.059	3	0.125	0.123	0.116	2	0.063	0.063	0.059	2	0.500	0.387	0.305	3	0.188	0.232	0.210	3	0.313	0.522	0.450	4	0.208	0.255	0.241
PasE300	1	0.000	0.000	0.000	1	0.000	0.000	0.000	1	0.000	0.000	0.000	2	0.313	0.272	0.229	1	0.000	0.000	0.000	2	0.188	0.175	0.155	2	0.083	0.080	0.077
PasE304	2	0.563	0.498	0.366	2	0.125	0.121	0.110	2	0.188	0.175	0.155	2	0.188	0.175	0.155	2	0.250	0.226	0.195	2	0.563	0.514	0.374	2	0.313	0.344	0.283
PasE348	2	0.563	0.498	0.366	3	0.875	0.534	0.412	1	0.000	0.000	0.000	3	0.625	0.494	0.431	3	0.625	0.486	0.416	2	0.188	0.175	0.155	4	0.479	0.426	0.392
PasE368	2	0.313	0.272	0.229	3*	0.875	0.573	0.456*	2*	0.000	0.484	0.359*	1	0.000	0.000	0.000	3	0.250	0.454	0.393	5*	0.313	0.518	0.477*	8	0.292	0.460	0.430
PasE380	3	0.750	0.599	0.511	2	0.438	0.353	0.283	3	0.375	0.411	0.354	3	0.313	0.506	0.397	3	0.625	0.615	0.522	2	0.500	0.508	0.371	3	0.500	0.582	0.488
PasE425	2	0.688	0.498	0.366	2	0.375	0.315	0.258	2	0.188	0.353	0.283	2	0.500	0.508	0.371	4	0.313	0.381	0.344	3	0.375	0.524	0.428	4	0.406	0.507	0.401
PasE447	1	0.000	0.000	0.000	2	0.188	0.175	0.155	2	0.625	0.484	0.359	2	0.563	0.417	0.323	2	0.063	0.063	0.059	2	0.188	0.272	0.229	3	0.271	0.272	0.245
PasE452	1	0.000	0.000	0.000	1	0.000	0.000	0.000	2	0.125	0.315	0.258	1	0.000	0.000	0.000	2	0.125	0.121	0.110	2	0.125	0.121	0.110	2	0.063	0.099	0.094
PasE480	1	0.000	0.000	0.000	2	0.063	0.063	0.059	2	0.063	0.063	0.059	2	0.125	0.121	0.110	2	0.188	0.175	0.155	2	0.063	0.063	0.059	2	0.083	0.080	0.077
PasE486	1	0.000	0.000	0.000	1	0.000	0.000	0.000	2	0.375	0.315	0.258	1	0.000	0.000	0.000	2	0.125	0.387	0.305	1	0.000	0.000	0.000	2	0.083	0.136	0.126
PasE487	2	0.225	0.265	0.200	2	0.375	0.315	0.258	2	0.313	0.498	0.366	2	0.063	0.063	0.059	2	0.250	0.226	0.195	2	0.375	0.315	0.258	2	0.229	0.306	0.258
Average	2.2	0.358	0.297	0.244	2.3	0.461	0.331	0.237	2.3	0.331	0.325	0.263	2.0	0.403	0.324	0.250	2.6	0.359	0.366	0.307	2.5	0.368	0.389	0.314	3.7	0.378	0.394	0.343

*Note*: *A* = number of alleles sampled; *H*_o_ = observed heterozygosity; *H*_e_ = expected heterozygosity; *N* = number of individuals sampled; PIC = polymorphism information content.

^a^ Voucher and locality information are provided in S1 Table.

* Significant deviation from Hardy-Weinberg equilibrium (P < 0.001).

At the level of the species, our results showed that total genetic diversity of *P*. *subaequalis* (*H*_T_) was 0.393 and genetic diversity within populations (*H*_S_) was 0.336 (S8 Table). Overall, *F*_ST_ and *G*_ST_ across the six natural populations of *P*. *subaequalis* were 0.171 and 0.147, representing a much higher genetic differentiation between populations. The AMOVA revealed that 16.74% of the total variation was attributed to differences among six populations and that 83.26% was contributed by differences within populations (*P* < 0.001; S9 Table), indicating the genetic variation of *P*. *subaequalis* mainly existed in individuals within populations. Besides, bottleneck analysis found only one population of *P*. *subaequalis* in Zhuxian Village of Anhui Province (ZXC) could have experienced the significant recent bottleneck under the three different models of microsatellite evolution (S10 Table).

## Discussion

### Characterization of the *Parrotia subaequalis* transcriptome using next-generation sequencing technologies

In recent years, the use of next-generation sequencing (NGS) technologies have become increasingly prevalent because of its high-throughput genomic and transcriptomic data output for model or non-model organisms at reasonable prices and schedules [34–36]. In the present study, we characterized the transcriptomes of two individuals of *P*. *subaequalis* using RNA-sequencing technology on the Illumina HiSeq 2500 platform for the first time. Raw data of these two transcriptomes are currently available to the public.

Approximately five Gb of data length for each individual of *P*. *subaequalis* were generated and assembled into unigenes. As a result, the mean length of the unigenes of *P*. *subaequalis* (TX) was 890 bp and 887 bp for *P*. *subaequalis* (SJD), suggesting that the large number of reads with paired-end information and high sequencing depth produced much longer unigenes than reported in previous transcriptome studies of *Neolitsea sericea* (mean length 733 bp) [37], *Sesamum indicum* (mean length 629 bp) [38] and *Pennisetum purpureum* (mean length 586 bp) [39].

In terms of the annotation of unigenes, the results showed a large part of unigenes (62.17% in *P*. *subaequalis* (TX) and 55.24% in *P*. *subaequalis* (SJD)) had homologs in public databases like Nr, Nt, InterPro, KOG, KEGG, Swiss-Port and GO. These annotated unigenes could provide valuable information for future studies on *P*. *subaequalis*. A minority of the unigenes (37.83% in *P*. *subaequalis* (TX) and 44.76% in *P*. *subaequalis* (SJD)) failed to match any proteins in the above public databases, which may be attributable to the large amount of short-length (< 500 nt) unigenes (Fig 2) or the limited publicly available genomic and transcriptomic information for *P*. *subaequalis*. Further explanations for the low hit possibility of short sequences were the lack of a characterized protein domain or the short query sequences [38], resulting in false-negative results. GO is a worldwide classification database for gene function; in our study, “metabolic process”, “cellular process”, “catalytic activity” and “binding” were the four most matched categories in two individuals of *P*. *subaequalis* (Fig 3 and S2 and S3 Tables). Additionally, KEGG analysis of the annotated unigenes showed that “global and overview maps”, “carbohydrate metabolism”, “translation”, “folding, sorting and degradation”, “amino acid metabolism” and “signal transduction” were the primary biological pathways in the two individuals of *P*. *subaequalis* (Fig 4 and S4 and S5 Tables). Overall, these findings here will greatly enrich the transcriptomic resources for further research on gene discovery, molecular mechanisms and biological pathways of *P*. *subaequalis*.

### Mining and utilization of polymorphic EST-SSR markers in conservation genetics

Prior to our study, molecular marker studies of *P*. *subaequalis* were conducted with ISSR, chloroplast SSR and nuclear SSR [6, 22], while no EST-SSR markers had been reported. EST-SSR markers are powerful molecular markers for analyzing population genetic diversity, cross transferability rate, molecular breeding and functions [40, 41]. With the wide application of the NGS technologies, the increasing number of transcriptome sequences have provided abundant resources for EST-SSR applications for research and genetic improvements. In addition, a number of bioinformatics software have been developed for SSR mining, such as MISA [42] and SSR Primer [43]. However, to date, these tools have not integrated a computational solution for systematic assessment of SSR polymorphic status, resulting in poor efficiency of polymorphic SSR identification and time-consuming experiments. The newly developed pipeline, CandiSSR, could help users detect candidate polymorphic SSRs with high efficiency [16]. Therefore, in the present study, using CandiSSR, we successfully and efficiently mined 497 candidate polymorphic EST-SSR markers from the two comparative transcriptomic datasets. Then, 54 randomly chosen primer pairs were used for validation of the polymorphism, and 27 primer pairs (50%) were proven to be polymorphic among 96 individuals from the six natural populations. Such high success ratios indicated that this kind of molecular development method with the aid of CandiSSR was highly efficient and considerably successful.

Among 497 candidate polymorphic EST-SSR markers, in agreement with previous reports from many other dicotyledonous plant taxa such as *Arabidopsis*, peanut, cabbage, pea, grape, soybean, sunflower [44], dinucleotide motifs (DNRs) were found to be the most frequent motif type (62.78%) in *P*. *subaequalis*, followed by TNRs (35.61%), TTRs (1.21%) and HNRs (0.40%) (Fig 5). Among the DNRs, AT/TA (37.18%) was quite dominant, followed by AG/TC (27.24%) and CT/GA (20.83%). CTG/AAG (10.17%) was the most abundant motif type for TNRs, followed by AGC/GCG (9.04%). Our results were consistent with previous reports on tree peony [45], radish [46] and sweet potato [47]. In our study, the GC/CG repeat motif was found in only 0.01% (Fig 5) of the dinucleotide repeats. As is well-known, a common feature in most dicotyledonous plants is the rarity of GC/CG in dinucleotide motifs [37, 44, 48], which was has been explained by the low GC content of dicotyledons [49].

Furthermore, using the 27 polymorphic EST-SSR markers, 100 alleles were found across the 96 individuals of *P*. *subaequalis* from six natural populations. The range of the number of alleles per locus was from 1 to 8 with a mean of 3.70 alleles, which was lower than the range from 2 to 14 and mean of 5.33 alleles in the gSSRs of *P*. *subaequalis* [6]. The average gene diversity (*H*_e_) and PIC value of the 27 polymorphic EST-SSR markers were 0.394 and 0.343, representing a moderate level of gene polymorphism compared to the gSSRs (mean: *H*_e_ = 0.558; PIC = 0.515) reported in Zhang et al. [6]. We observed a considerably higher level of transferability (92.59%) in five congeneric Hamamelidaceae species than the gSSR (66.67%) reported by Zhang et al. [6]. The much higher level of cross-transferability and the slightly lower degree of gene polymorphism of EST-SSRs than of gSSRs reflected the highly conserved character of the flanking sequences of EST-SSRs and the low mutation frequency of EST sequences. Additionally, our EST-SSR survey of six natural population of *P*. *subaequalis* revealed a relatively high level of genetic diversity (*H*_T_ = 0.393; *H*_S_ = 0.336; S8 Table) and a little higher genetic differentiation level (*F*_ST_ = 0.171; S8 Table) at the level of species, suggesting *P*. *subaequalis* maintained high levels of species diversity in the long-term evolutionary history despite its restricted and highly disjunct distribution range. The observation of genetic diversity and bottleneck test among six wild *P*. *subaequalis* populations indicated that WFS was the most variable population, while SJD and ZXC was the two more endangered populations that we should pay more attention to their protection and preservation. The Wangfo Mountain (WFS) was considered as one of the biodiversity refugia since the Tertiary in China [50, 51] and few human activities were found there, thus contributing the highest genetic diversity in WFS population in some degree. While based on our field observations, SJD population was located in the scenic area of Shanjuan Cave and the population ZXC lied in a village, the human interference including farming and foresting may result in the lower level of genetic diversity and recent bottleneck. In summary, the polymorphic EST-SSR markers developed here will provide a powerful tool for further studies on conservation genetics and molecular breeding of *P*. *subaequalis* and other Hamamelidaceae species.

## Conclusions

This study is the first to assemble and characterize the transcriptomes of two individuals of *P*. *subaequalis* using RNA-sequencing technologies on the Illumina HiSeq 2500 platform. This large set of annotated unigenes and pathways will remarkably enlarge the transcriptomic resources and putative gene functions of *P*. *subaequalis*. In addition, we successfully and efficiently developed the first set of 27 novel polymorphic EST-SSR markers for *P*. *subaequalis* from the two transcriptomic datasets. These polymorphic EST-SSR markers displayed a relatively high genetic diversity in *P*. *subaequalis* and high transferability in five related Hamamelidaceae species, suggesting that they are useful and powerful molecular tools to facilitate future studies on population genetics, molecular breeding and germplasm identification of *P*. *subaequalis* and other Hamamelidaceae species. Taken together, these results produced by our study indicated that high-throughput next-generation sequencing technology is a cost-effective and convenient approach to mining abundant novel molecular resources for non-model organisms.

## Supporting information

S1 Table Locality and voucher information for populations of *Parrotia subaequalis* and the Hamamelidaceae species used in this study. (DOCX)

S2 Table Go classification of *Parrotia subaequalis* (TX) unigenes. (XLS)

S3 Table Go classification of *Parrotia subaequalis* (SJD) unigenes. (XLS)

S4 Table KEGG classification for unigenes of *Parrotia subaequalis* (TX). (XLS)

S5 Table KEGG classification for unigenes of *Parrotia subaequalis* (SJD). (XLS)

S6 Table The candidate polymorphic EST-SSRs of two individuals of *Parrotia subaequalis*. (XLS)

S7 Table The primer pairs of candidate polymorphic EST-SSRs of two individuals of *Parrotia subaequalis* and 54 primer pairs (highlighted in the table) selected for the polymorphism validation and transferability tests. (XLS)

S8 Table Genetic diversity of the 27 polymorphic EST-SSR loci for *Parrotia subaequalis*. (DOCX)

S9 Table Analysis of molecular variance (AMOVA) within/among six *P*. *subaequalis* populations using EST-SSR markers. (DOCX)

S10 Table Bottleneck detection for six natural populations of *P*. *subaequalis*. (DOCX)
