# Supplementary material for: Mining and characterization of novel EST-SSR markers of Parrotia subaequalis (Hamamelidaceae) from the first Illumina-based transcriptome datasets
Source: PLoS One. 2019 May 6;14(5):e0215874. doi: 10.1371/journal.pone.0215874 (PMC6502335; doi:10.1371/journal.pone.0215874)
Supplement: S1 Table — (DOCX) [file pone.0215874.s001.docx]

Table S1. Locality and voucher information for populations of *Parrotia subaequalis* and the Hamamelidaceae species used in this study.

| Species | Population code | Voucher specimens^a^ | Collection locality | Geographic coordinates | Altitude (m) | *N* |
| --- | --- | --- | --- | --- | --- | --- |
| *Parrotia subaequalis* | SJD | Yunyan Zhang, ZYY16082401 | Shanjuan Cave, Jiangsu Province, China | 31.5806N, 119.8004E | 312-485 | 16 |
| *Parrotia subaequalis* | HBS | Yunyan Zhang, ZYY16091806 | Mt. Huangbo, Henan Province, China | 33.4340N, 116.0471E | 682-899 | 16 |
| *Parrotia subaequalis* | TX | Yunyan Zhang, ZYY16083002 | Mt. Tianxia, Anhui Province, China | 30.8918N, 116.3617E | 417-522 | 16 |
| *Parrotia subaequalis* | ZXC | Yunyan Zhang, ZYY16090805 | Zhuxian Village, Anhui Province, China | 30.1259N, 118.5405E | 718 | 16 |
| *Parrotia subaequalis* | WFS | Yunyan Zhang, ZYY16082903 | Mt. Wangfo, Anhui Province, China | 32.1492N, 117.4469E | 461-687 | 16 |
| *Parrotia subaequalis* | LWS | Yunyan Zhang, ZYY16090704 | Mt. Longwang, Zhejiang Province, China | 30.2454N, 119.2484E | 876-1238 | 16 |
| *Parrotia persica* | ─ | Pan Li, LP174414 | Chenshan Botanical Garden, Shanghai, China | 31.4545N, 121.1038E | 17 | 5 |
| *Parrotiopsis jacquemontana* | ─ | Pan Li, LP174412 | Zurich University Botanical Gardens, Switzerland | 47.2229N, 8.3255E | 435 | 5 |
| *Sycopsis sinensis* | ─ | Pan Li, LP172890 | Hangzhou Botanical Garden, Zhejiang Province, China | 30.1514N, 120.7171E | 18 | 5 |
| *Distylium racemosum* | ─ | Pan Li, LP172887 | Hangzhou Botanical Garden, Zhejiang Province, China | 30.1514N, 120.7171E | 20 | 5 |
| *Hamamelis virginiana* | ─ | Pan Li, CB08834 | Mt. Wujia Forest Farm, Hubei Province, China | 31.0426N, 116.1534E | 931 | 5 |

*Note: N* = number of individuals sampled; ^a^ Vouchers were deposited in the Herbarium of Zhejiang University (HZU), Hangzhou, Zhejiang Province, China.
