# Supplementary material for: Mining and characterization of novel EST-SSR markers of Parrotia subaequalis (Hamamelidaceae) from the first Illumina-based transcriptome datasets
Source: PLoS One. 2019 May 6;14(5):e0215874. doi: 10.1371/journal.pone.0215874 (PMC6502335; doi:10.1371/journal.pone.0215874)
Supplement: S8 Table — (DOCX) [file pone.0215874.s008.docx]

Table S8. Genetic diversity of the 27 polymorphic EST-SSR loci for *Parrotia subaequalis*^a^.

| Locus | *H*_T_ | *H*_S_ | *F*_ST_ | *G*_ST_ |
| --- | --- | --- | --- | --- |
| PasE6 | 0.529 | 0.340 | 0.401 | 0.358 |
| PasE20 | 0.575 | 0.531 | 0.090 | 0.076 |
| PasE27 | 0.469 | 0.432 | 0.094 | 0.080 |
| PasE83 | 0.585 | 0.457 | 0.251 | 0.218 |
| PasE108 | 0.220 | 0.213 | 0.037 | 0.031 |
| PasE156 | 0.612 | 0.490 | 0.231 | 0.200 |
| PasE159 | 0.590 | 0.488 | 0.201 | 0.173 |
| PasE178 | 0.611 | 0.523 | 0.169 | 0.145 |
| PasE180 | 0.664 | 0.614 | 0.090 | 0.076 |
| PasE188 | 0.504 | 0.466 | 0.089 | 0.075 |
| PasE198 | 0.061 | 0.060 | 0.030 | 0.025 |
| PasE205 | 0.162 | 0.115 | 0.332 | 0.293 |
| PasE208 | 0.577 | 0.468 | 0.220 | 0.190 |
| PasE218 | 0.433 | 0.411 | 0.060 | 0.051 |
| PasE268 | 0.485 | 0.431 | 0.131 | 0.112 |
| PasE290 | 0.255 | 0.232 | 0.103 | 0.087 |
| PasE300 | 0.080 | 0.074 | 0.087 | 0.074 |
| PasE304 | 0.343 | 0.284 | 0.200 | 0.172 |
| PasE348 | 0.425 | 0.361 | 0.176 | 0.151 |
| PasE368 | 0.460 | 0.386 | 0.186 | 0.160 |
| PasE380 | 0.582 | 0.499 | 0.167 | 0.143 |
| PasE425 | 0.507 | 0.431 | 0.176 | 0.151 |
| PasE447 | 0.271 | 0.234 | 0.161 | 0.138 |
| PasE452 | 0.099 | 0.094 | 0.067 | 0.057 |
| PasE480 | 0.080 | 0.081 | 0.004 | 0.004 |
| PasE486 | 0.136 | 0.118 | 0.154 | 0.132 |
| PasE487 | 0.306 | 0.236 | 0.262 | 0.228 |
| Mean | 0.393 | 0.336 | 0.171 | 0.147 |

*Note:* *H*_T_ = Total genetic diversity for the species; *H*_S_ = Genetic diversity within populations; *F*_ST_ = Interpersonal inbreeding coefficient; *G*_ST_ = Gene differentiation factor. ^a^ Voucher and locality information are provided in Table S1.
