# Supplementary material for: Mining and characterization of novel EST-SSR markers of Parrotia subaequalis (Hamamelidaceae) from the first Illumina-based transcriptome datasets
Source: PLoS One. 2019 May 6;14(5):e0215874. doi: 10.1371/journal.pone.0215874 (PMC6502335; doi:10.1371/journal.pone.0215874)
Supplement: S9 Table — (DOCX) [file pone.0215874.s009.docx]

Table S9. Analysis of molecular variance (AMOVA) within/among six *P. subaequalis* populations using EST-SSR markers.

| SSR type | Source of variation | *d*_f_ | SSD | Variance components | Percentage variation | *P*-value |
| --- | --- | --- | --- | --- | --- | --- |
| EST-SSR | Among populations | 5 | 169.208 | 0.91531 | 16.74% | < 0.001 |
|  | Within populations | 186 | 846.625 | 4.55175 | 83.26% | < 0.001 |

*Note*: *d*_f_ = degrees of freedom; SSD = sum of squared deviation; *P*-value = probability.
