# Supplementary material for: Mining and characterization of novel EST-SSR markers of Parrotia subaequalis (Hamamelidaceae) from the first Illumina-based transcriptome datasets
Source: PLoS One. 2019 May 6;14(5):e0215874. doi: 10.1371/journal.pone.0215874 (PMC6502335; doi:10.1371/journal.pone.0215874)
Supplement: S10 Table — (DOCX) [file pone.0215874.s010.docx]

Table S10. Bottleneck detection for six natural populations of *P. subaequalis*.

| Population Code |  | Wilcoxon test |  |
| --- | --- | --- | --- |
|  | IAM | TPM | SMM |
| SJD | 0.104 | 0.465 | 0.768 |
| HBS | 0.061 | 0.234 | 0.560 |
| TX | 0.086 | 0.622 | 0.540 |
| ZXC | 0.002^**^ | 0.010^*^ | 0.049^*^ |
| WFS | 0.148 | 0.771 | 0.542 |
| LWS | 0.025^*^ | 0.291 | 0.617 |

*Note*: IAM = Infinite allele model; TPM = Two-phased mutation model; SMM = Stepwise mutation model.

Significant level: ^*^ P < 0.05; ^**^ P < 0.01; ^***^ P < 0.001.
